# Effect of a participatory organizational-level occupational health intervention on job satisfaction, exhaustion and sleep disturbances: results of a cluster randomized controlled trial

**DOI:** 10.1186/s12889-016-3871-6

**Published:** 2016-11-29

**Authors:** Elisabeth Framke, Ole Henning Sørensen, Jacob Pedersen, Reiner Rugulies

**Affiliations:** 1 Center for Industrial Production, Aalborg University Copenhagen, A. C. Meyers Vænge 15, DK-2450 Copenhagen, Denmark; 2 National Research Centre for the Working Environment, Lersø Parkallé 105, DK-2100 Copenhagen, Denmark; 3 Department of Public Health, University of Copenhagen, Øster Farimagsgade 5, DK-1014 Copenhagen, Denmark; 4 Department of Psychology, University of Copenhagen, Øster Farimagsgade 2A, DK-1353 Copenhagen, Denmark

**Keywords:** Psychosocial, Stress-as-Offense-to-Self, Core task, Occupational health, Well-being at work

## Abstract

**Background:**

We examined whether the implementation of a participatory organizational-level intervention aiming to improve the working environment with a focus on the core task at work, increased job satisfaction and reduced exhaustion and sleep disturbances among pre-school employees.

**Methods:**

The study sample consisted of 41 intervention group pre-schools with 423 employees and 30 control group pre-schools with 241 employees. The intervention lasted 25 months and consisted of seminars, workshops, and workplace specific intervention activities that were developed by focusing on the core task at work. We analyzed within-group changes in the three outcome variables from baseline to follow-up with t-tests for paired samples, separately for intervention and control group. Between-group differences in changes in the three outcome variables were analyzed using a mixed model with a repeated statement to account for the clustering effect of workplaces.

**Results:**

Within-group analyses showed that exhaustion decreased statistically significantly in both the intervention and the control group. There were no statistically significantly changes in job satisfaction and sleep disturbances. Between-group analyses showed that there was no statistically significant difference between the two groups for changes in any of the outcome variables, neither in the unadjusted or in the adjusted analyses.

**Conclusions:**

We found no evidence that participating in an organizational-level occupational health intervention aiming to improve the working environment with a focus on the core task at work has an effect on pre-school employees’ job satisfaction, exhaustion and sleep disturbances.

**Trial registration:**

ISRCTN16271504, November 15, 2016.

## Background

The relation of the psychosocial work environment with employees’ health and well-being is likely highly complex and characterized by many factors [1]. Adverse psychosocial working conditions that had been related to health endpoints include for example mismatches between high demands and low control [2–4], and high effort and low reward [5, 6], poor management style [7] and organisational injustice [8]. Psychosocial resources at work, such as high workplace social capital [9–11] may contribute to the protection of employees’ health. Organizational-level occupational health interventions aim for reducing health-hazardous and enhancing health promoting working conditions [12]. It has been argued, that in particular participatory organizational interventions may have a positive impact on employees’ health, partly because these types of interventions improve employees’ job control [13]. The participatory approach refers to employees’ involvement and participation and implies that employees participate in workplace problem analysis and take an active part in developing and implementing intervention activities tailored their own workplace [14, 15]. However, results from organizational interventions are inconsistent and study quality is often low [16, 17].

According to the Stress-As-Offense-to-Self (SOS) theory, the distinction between core tasks and illegitimate tasks at work are key for understanding employees’ health and well-being [18]. Core tasks are activities that are essential for fulfilling the purpose of the organization and are closely linked to the professional identity of an employee. For a nurse, for example, it is a core task to take care of the medical needs of a patient. In the SOS theory, illegitimate work tasks are defined as the opposite of core work tasks and regarded as stressors, potentially affecting employees’ health and well-being. They are conceptualized as either unnecessary, i.e. they should not be done at all or as unreasonable, i.e. they are outside one’s occupation or occupational status and should be done by others. Previous research has shown that carrying out illegitimate tasks, as opposed to core tasks, is associated with counterproductive work behavior [19], higher level of cortisol [20], elevated stress level [21], decreased mental health [22], sleep disturbances [23], lowered self-esteem [18, 24] and feelings of resentment towards ones organization and burnout [18].

In this article, we evaluate the effect of a participatory organizational intervention that aimed to improve the working environment with a focus on the core task at work. In a previous article, we had shown that the intervention predicted a lower risk of sickness absence in the intervention group compared to the control group [25]. In this article, we test the effect of the intervention on three variables: job satisfaction, exhaustion and sleep disturbances. We hypothesized that the intervention will lead to increased job satisfaction and reduced exhaustion and sleep disturbances in intervention group participants compared to control group participants.

The hypothesis is built on the underlying assumption that a psychosocial workplace intervention focusing on the core tasks at work will reduce exposure to adverse psychosocial working conditions, i.e. work stressors, and that reduced exposure to work stressors will result in more job satisfaction and less exhaustion and sleep disturbances. We choose job satisfaction as a general measure of employees’ well-being at work, as suggested in previous studies, for example Bond and Bunce [26], Pryce et al [27], and DeJoy et al [28]. There is a strong relationship between job satisfaction and health, in particular for aspects of mental health [29]. Exhaustion and sleep disturbances are important health problems that are suspected to be at least partly related to the work environment [30, 31]. Further, exhaustion is a core symptom of the burnout syndrome that is common among human service workers and is a key topic in work environment research [32]. Previous research concluded that burnout increases the likelihood of sickness absence [33], and suggested that changes in the psychosocial work environment can reduce risk of burnout [34]. The relationship between psychosocial working conditions and exhaustion was also found in other studies [31, 35]. With regard to sleep disturbances, a recent review showed that psychosocial work factors impact sleep disturbances and called for work environment intervention studies tackling sleep disturbances [36].

## Methods

The aim of this intervention study was to study municipal pre-schools. The intervention was an initiative from the Municipality of Copenhagen, Denmark. The intervention was implemented in pre-schools in the Children and Youth Administration in Copenhagen by eight professional working environment consultants from a private company. The research evaluation was conducted by the University of Aalborg and the Danish National Research Centre for the Working Environment (NRCWE).

### Study design and participants

This is an organizational-level occupational health intervention study that was cluster randomized and parallel with two arms. Questionnaire measurements were conducted at baseline and at 24 months of follow-up. Seventy eight workplaces formed the cluster randomized controlled trial. The Municipality of Copenhagen decided to conduct the intervention at 44 pre-schools, whereas 34 pre-schools served as the control group. A statistician randomized the workplaces accordingly. Of the 44 intervention workplaces, three were lost during follow-up: one workplace did not receive the intervention, because the workplace was preoccupied with other project activities; two workplaces discontinued the intervention, one was closed during follow-up and one left the study because the management had a negative appraisal of the intervention. Of the 34 control group workplaces, four were lost because they did not provide baseline or follow-up measurements. Thus, the analyses were based on 41 intervention and 30 control group workplaces

**Fig. 1:**
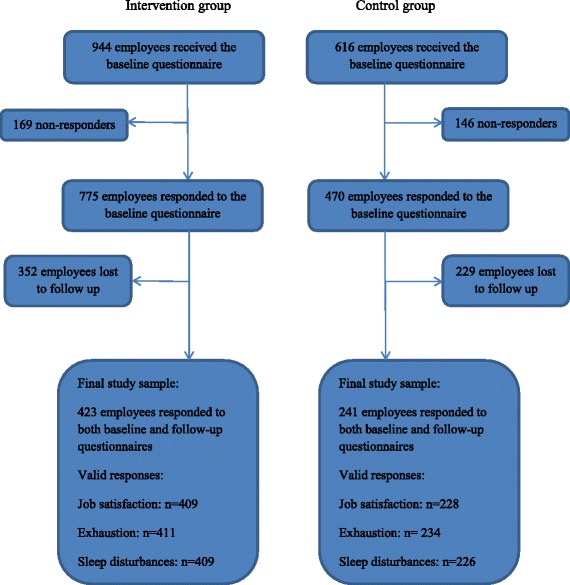
Flow chart towards the final study sample

Employees were eligible for the study if they were employed and present at the intervention and control group workplaces during the time of the baseline questionnaire measurements. Figure 1 shows that at baseline (September 2011), 944 employees at the intervention group pre-schools and 616 employees at the control group pre-schools received the questionnaire. Of these, 775 in the intervention (82.1%) and 470 (76.3%) in the control group responded. Of the 775 intervention group baseline responders, 423 employees responded to the follow-up questionnaire 24 months later, whereas 352 employees were lost to follow up. Due to missing information on some of the outcome measures, the final study sample in the intervention group was *n* = 409 for job satisfaction, *n* = 411 for exhaustion, and *n* = 409 for sleep disturbances. Of the 470 control group baseline responses, 241 employees responded to the follow-up questionnaire, whereas 229 were lost to follow up. The final study sample in the control group was *n* = 228 for job satisfaction, *n* = 234 for exhaustion, and *n* = 226 for sleep disturbances. According to Danish law, research studies that use solely questionnaire and register data do not need approval from the National Committee on Health Research Ethics (Den Nationale Videnskabetiske Komité).

### The intervention

The intervention was a participatory intervention focusing on the core task at work. Employees participated in the workplace problem analysis and solution finding process to ensure employees’ involvement, commitment and control and at the same time to ensure that intervention activities were tailored to the specific needs of the workplace. In each intervention workplace, the pedagogical leader and two employee representatives, the shop steward and the health and safety representative, formed a steering group that managed the intervention. Each steering group received implementation support from a professional working environment consultant for the full intervention period. The intervention consisted of intervention activities common for all steering groups, i.e. seminars and workshops on how to develop and implement workplace specific intervention activities using a participatory approach, change management, workplace culture and evaluation tools. Based on the common intervention activities and consultants implementation support, the steering groups developed and implemented workplace specific intervention activities involving all employees. The intervention followed a structured and step-wise approach. From September 2010 to September 2011, the intervention project leader team planned and coordinated the intervention study. For five months from September 2011, workplace specific intervention activities were developed by the steering groups with the participation of all employees. Consultants explained to workplaces that this intervention’s focus on the core task at work was equivalent to develop activities to improve the performance of central work tasks and procedures. The implementation lasted from February 2012 to June 2013. Finally, the workplaces conducted a self-evaluation between March and June 2013.

### Effect measures

We measured effects on changes in job satisfaction, exhaustion and sleep disturbances with self-administered questionnaires at baseline and at 24 months of follow-up. Both intervention and control group employees received and responded to the questionnaires during working hours.

We measured job satisfaction with one item (Regarding your work in general. How satisfied are you with your job as a whole, everything taken into consideration?’), rated on a four-point scale (very satisfied, satisfied, dissatisfied, very dissatisfied) [37]. We measured exhaustion (’Within the past two weeks, how much of the time have you felt lacking in energy and strength?’) and sleep disturbances (’Within the past two weeks, how much of the time have you had trouble sleeping at night?’) with one item each, derived from the Major Depression Inventory [38]. Responses were rated on a six-point scale (all of the time, most of the time, slightly more than half of the time, slightly less than half of the time, some of the time, at no time). Higher scores indicate more job satisfaction, more exhaustion and more sleep disturbances.

### Statistical analysis

All analyses were conducted using SAS 9.3.

First, to test baseline differences between intervention and control group in the study sample, we used Chi-square test, two sample t-test and Proc GLM.

Next, we calculated the baseline and follow-up mean scores for each outcome variable separately for the intervention and the control group. Using paired t-tests, we analyzed changes from baseline to follow-up for each outcome variable, separately within the intervention and within the control group.

Next, using the Genmod procedure in SAS, we analyzed differences in changes of the outcome variables between the intervention and the control group during follow-up in a mixed model with a repeated statement to account for the clustering effect of workplaces.

We calculated unadjusted estimates and estimates adjusted for sex and age (continuous) (Model 1) and further adjusted for job group (pedagogical leader, nursery nurse, nursery nurse assistant, other job group), workplace type (integrated, day care, kindergarten) and workplace size (continuous) (Model 2).

Finally, we conducted post-hoc analyses, in which we repeated the between group analyses while adjusting for the baseline scores of the outcome variables.

## Results

### Characteristics of participants

Table 1 shows employee and workplace characteristics in the intervention and control group. Compared to control group participants, intervention group participants were younger (mean age: 42.9 vs. 44.9 years, *p* = 0.02) and were employed at workplaces of greater size (mean size 23.4 vs. 21.8 employees, *p* = 0.02). The groups did not differ with regard to sex, job group, and workplace type.

**Table 1:** Employee and workplace characteristics and baseline scores of outcome variables in the intervention and the control group in the study sample

	Intervention group				Control group				Chi^2^ (p)	t (p)
	Mean	SD	%	*n*	Mean	SD	%	*n*		
**Employee characteristic**				423				241		
Age	42.9	10.4			44.9	9.8				**2.43 (0.02)**
Women			87.0	368			90.0	217	1.36 (0.24)	
Job group									2.05 (0.56)	
- Pedagogical leaders			6.6	28			6.2	15		
- Nursery nurses			57.2	242			52.7	127		
- Nursery nurse assistants			28.4	120			30.7	74		
- Other job groups			7.8	33			10.4	25		
**Workplace characteristics**				41				30		
Size	23.4	8.4			21.8	9.6				**-2.25 (0.02)**
Workplace type									2.26 (0.32)	
- Integrated			77.1	326			79.7	192		
- Day care			18.7	79			18.3	44		
- Kindergarden			4.3	18			2.1	5		
**Baseline scores of outcome variables**										
Job satisfaction	3.19	0.57		409	3.02	0.70		228		**-3.11 (0.002)**
Exhaustion	2.72	1.13		411	3.01	1.26		234		**3.06 (0.002)**
Sleep disturbances	2.04	1.28		409	2.34	1.40		226		**2.68 (0.008)**

Statistically significant results are printed in **bold**

Table 1 also shows the baseline scores of the three outcome variables. Intervention group participants had more favorable scores on all three variables, i.e. they reported more job satisfaction and less exhaustion and sleep disturbances than control group participants. These differences remained statistically significant, when we adjusted the analyses for employee and workplace characteristics (age, sex, job group, workplace size and workplace type, data not shown but is available on request from the first author).

### Comparison of the study sample with employees lost during follow-up

When comparing the participants in the study sample with the participants that dropped out during follow-up, we found that the drop-out pattern was similar in the intervention and control group with regard to age and sex. In both groups, younger employees compared to older employees and men compared to women were more likely to drop out of the study. In the intervention group, mean age was 39.7 years for dropouts and 42.9 years for non-dropouts (*p* < 0.0001) compared to 41.1 years for dropouts and 44.9 years for non-drop-outs in the control group (*p* = 0.0002). Proportion of men was 17.9% among dropouts and 13.0% among non-dropouts in the intervention group (*p* = 0.06) and 16.2% among dropouts and 10.0% among non-dropouts in the control group (*p* = 0.05). In addition, in the intervention group, the mean workplace size was 26.0 employees among those who dropped out of the study compared to 23.4 employees among employees in the study sample (*p* < 0.0001). There was no such pattern in the control group (workplace size: 22.1 vs. 21.8 for those who dropped out and remained, respectively, *p* = 0.71). In both intervention and control group, participants who dropped out had a higher exhaustion score at baseline compared to those who remained in the study. This difference was statistically significant in the intervention group (2.93 vs. 2.72, *p* = 0.01) but not in the control group (3.16 vs. 3.01, *p* = 0.20).

### Effect of the intervention on job satisfaction, exhaustion and sleep disturbances

Table 2 shows within group changes from baseline to follow-up in job satisfaction, exhaustion and sleep disturbances. Exhaustion decreased statistically significantly in both the intervention group (-0.16 points, *p* = 0.01) and the control group (-0.29 points, *p* < 0.001). There was no statistically significant change in job satisfaction and sleep disturbances, neither in the intervention group nor the control group.

**Table 2:** Within group changes in job satisfaction, exhaustion and sleep disturbances during 24 months of follow-up

	Intervention group (*n* = 423)						Control group (*n* = 241)					
	*n*	Baseline Mean (SD)	Follow-up Mean (SD)	change	t	*p*	*n*	Baseline Mean (SD)	Follow-up Mean (SD)	change	T	*p*
Job satisfaction	409	3.19 (0.57)	3.20 (0.54)	+0.01	0.29	0.77	228	3.02 (0.70)	3.09 (0.62)	+0.07	1.21	0.23
Exhaustion	411	2.72 (1.13)	2.56 (1.17)	-0.16	-2.50	**0.01**	234	3.01 (1.26)	2.73 (1.16)	-0.29	-3.48	**<0.001**
Sleep disturbances	409	2.04 (1.28)	1.97 (1.22)	-0.08	-1.14	0.26	226	2.34 (1.40)	2.25 (1.36)	-0.09	-0.94	0.35

Statistically significant results are printed in **bold**

Table 3 shows the between-group changes for job satisfaction, exhaustion and sleep disturbances. There was no statistically significant difference between the intervention and control group for any of the three variables, neither in the crude nor in the adjusted analyses.

**Table 3:** Intervention effect on job satisfaction, exhaustion and sleep disturbances in the intervention group compared to the control group during 24 months of follow-up

	*n*	Unadjusted			Model 1			Model 2		
		Est	95% CI	*p*	Est	95% CI	*p*	Est	95% CI	*p*
Job satisfaction	637	-0.06	-0.21–0.10	0.47	-0.04	-0.20–0.11	0.59	-0.06	-0.21–0.09	0.40
Exhaustion	645	0.13	-0.12–0.37	0.31	0.12	-0.12–0.37	0.31	0.15	-0.08–0.38	0.20
Sleep disturbances	635	0.01	-0.28–0.31	0.93	0.01	-0.29–0.31	0.94	0.03	-0.24–0.31	0.82

Interaction change x group analyses: Unadjusted; Model 1: Adjusted for sex and age (continuous); Model 2: further adjusted for job group (pedagogical leader, nursery nurse, nursery nurse assistant, other job group), workplace type (integrated, day care, kindergarden) and workplace size (continuous). Workplace identification number is included in a repeated statement

### Post-hoc analyses

Because we had found that intervention and control group differed statistically significantly in the baseline scores of the three outcome variables (see Table 1), we conducted post-hoc analyses that repeated the between-group analyses in Table 3, while adjusting for the baseline values of the outcome variables. The estimates from this post-hoc analyses were similar to the estimates reported in Table 3 (data not shown but is available on request from the first author).

## Discussion

The hypothesis that this intervention, which was a participatory organizational-level intervention aiming to improve the working environment with a focus on the core task at work, would improve job satisfaction and reduce exhaustion and sleep disturbances was not confirmed. There were no statistically significant differences between the intervention and control group during a 24 months follow-up.

In a previous article of the same intervention study, we had shown that intervention group participants had a decreased risk of sickness absence during follow-up compared to control group participants [25]. Taken the previous and the current finding together, it seems that the intervention was efficacious with regard to sickness absence but not with regard to job satisfaction, exhaustion and sleep disturbances. However, one has to be cautious with drawing conclusions by comparing the two analyses, because the two samples were only partly overlapping. In the analysis on sickness absence, we used register data to assess the outcome variable and therefore we were able to analyze sickness absence for all employees at all workplaces, including employees who left the workplace during follow-up (who were excluded on the day they left their workplace) and employees who newly started at a workplace during follow-up (who were included on the day, they entered the workplace) [25]. In the current analysis on job satisfaction, exhaustion and sleep disturbances, register data was not available and therefore the analysis was restricted to employees who filled in the questionnaire at both baseline and follow-up. Moreover, sickness absence was assessed with monthly updates throughout the whole follow-up period, whereas job satisfaction, exhaustion and sleep disturbances were only assessed twice, at baseline and at the follow-up measure after 24 months.

When an intervention study failed to show an impact of the intervention, two main explanations have to be considered: theory failure or implementation failure [39]. Theory failure refers to that the theory was wrong. In the case of this study, this would mean that the theoretical assumption was wrong that a participatory organizational-level intervention aiming to improve the working environment with a focus on the core tasks at work would result in less job stress, which subsequently would result in more job satisfaction and less exhaustion and sleep disturbances. Implementation failure refers to that the theory was correct, but that the intervention was not appropriately implemented and that therefore the impact of the intervention could not be demonstrated.

We cannot decide whether theory or implementation failure or other mechanisms are the most likely explanations for the null findings. A previous qualitative process evaluation of the implementation of the intervention at four selected workplaces [40] showed that the four workplaces implemented specific intervention activities to solve organizational and professional conditions that were necessary to improve the performance of the core task. Thus, this qualitative process evaluation indicates that the intervention was appropriately implemented in at least some workplaces. In addition, the effect on risk of sickness absence [25] suggests that implementation failure is not likely.

Both the intervention and the control group showed a statistically significant reduction in exhaustion. We cannot rule out that the reduction in the control group was partly an effect of the intervention, if we assume that intervention knowledge has been spread from intervention group pre-schools to control group pre-schools. Such a contamination was theoretically possible as there was contact and exchange, including meetings, between managers and employees’ representatives of the intervention and control group pre-schools. However, if contamination actually had happened and if this contamination explains the reduction in exhaustion in the control group we do not know.

In addition to theory or implementation failure, methodological issues also may be an explanation of the null findings. At baseline, there was a highly significant difference between the intervention and control group in all three outcome variables, with the intervention group showing more job satisfaction and less exhaustion and sleep disturbances. These differences could not be explained by different employee or workplace characteristics in intervention and control group. Because of these differences in baseline scores of the outcome variables, it was more difficult for the intervention group than for the control group to show improvements during follow-up. We do not have a clear explanation why the two groups differed at baseline. One possible explanation is that this was due to chance as this was a cluster- and not an individual-randomized trial with only 78 clusters. Another explanation could be the setting when the baseline questionnaire was filled in. Intervention and control group participants filled in the questionnaire after they had been informed about the result of the randomization and it is possible that this has resulted in a better mood in the intervention group compared to the control group, which may have caused reporting of more job satisfaction and less exhaustion and sleep disturbances.

It seems that attrition rate was higher among younger employees than older employees and among those with high levels of exhaustion at baseline compared to those with low levels of exhaustion. This might have led to underestimation of the intervention effect, if the intervention was particularly efficacious for younger employees and those with high levels of exhaustion. Conversely, an intervention effect would have been overestimated if the intervention was particularly non-efficacious among younger employees and among those with high levels of exhaustion.

Strengths of the study are the cluster-randomized design and the comprehensive, structured and step-wise intervention approach. Response rate at baseline was high in both intervention and control group. Limitations of this study, in addition to that intervention and control group participants filled in questionnaires after randomization, were the use of single items to measure outcome variables and the rather long follow-up period. By measuring each outcome variable with one question only, we only measured limited aspects of job satisfaction, exhaustion and sleep disturbances. It is possible that results would have been different, if we had measured these three variables more comprehensively. Finally, 24 months is a rather long follow-up period, which was mainly due to that the intervention itself was conducted over a longer period that lasted, at least in some pre-schools, from September 2011, when the first intervention activities were planned, until June 2013, when the last intervention activities had been implemented. It is possible that there were effects at some point during the follow-up period that did not remain after 24 months, but also possible that some effects only occurred at the end of the intervention. In hindsight, it would have been better, if we would have assessed the endpoints more frequently, for example at 6, 12, 18 and 24 months of follow-up. This would have allowed us to more closely monitor how trajectories in health and well-being changed in relation to the intervention.

## Conclusion

We found no evidence that participating in an organizational-level occupational health intervention aiming to improve the working environment focusing on the core task at work has an effect on pre-school employees’ job satisfaction, exhaustion and sleep disturbances.

## Abbreviations

NRCWE: The Danish National Research Centre for the Working Environment
SOS: Stress-As-Offense-to-Self
